# Soil organic matter and CO_2_ fluxes in small tropical watersheds under forest and cacao agroforestry

**DOI:** 10.1371/journal.pone.0200550

**Published:** 2018-07-16

**Authors:** Eline Nayara Dantas da Costa, Marcelo Friederichs Landim de Souza, Paulo Cesar Lima Marrocos, Dan Lobão, Daniela Mariano Lopes da Silva

**Affiliations:** 1 Department of Biological Sciences, Universidade Estadual de Santa Cruz, Ilhéus, Bahia, Brazil; 2 Department of Exact and Technological Sciences, Universidade Estadual de Santa Cruz, Ilhéus, Bahia, Brazil; 3 Cocoa Research Center, Comissão Executiva do Plano da Lavoura Cacaueira, Ilhéus, Bahia, Brazil; Pacific Northwest National Laboratory, UNITED STATES

## Abstract

Annual estimates of CO_2_ and dissolved carbon concentrations in the soil profile provide valuable insight into the dynamics of organic matter in soil and the effect of changes to vegetation cover. The aim of this study was to observe the spatial influence of litter decomposition in the first few centimeters of the soil for CO_2_ fluxes and to describe the processing of soil organic matter throughout the soil profile by comparing three small tropical watersheds. Data were collected biweekly for six months, from December 2015 to May 2016. CO_2_ was measured using an infrared gas analyzer in fixed chambers and the dissolved carbon of soil solution was analyzed in a TOC analyzer. No differences were found in the total soil CO_2_ fluxes (control flux treatments) between the three study areas. In both cacao agroforestry systems (managed and unmanaged), total CO_2_ fluxes were influenced by the decomposition of litter. CO_2_ emissions in the soil profile of the cacao agroforestry systems were highly variable, compared to the preserved forest, and highly dependent on the soil characteristics attributed to the type of vegetation cover. Although a definite pattern between the temperature and soil moisture was not identified, these parameters showed a strong relationship in controlling the release of CO_2_ between treatments. The organic and inorganic dissolved carbon patterns in the soil solution of the three areas revealed different responses of soil organic matter processing related to soil characteristics and vegetation. The results confirm the hypothesis that the top of soils (total CO_2_ fluxes) of both cacao agroforestry systems (managed and unmanaged) emits fluxes of CO_2,_ which do not differ statistically from the preserved forest. However, depending on the soil characteristics, the cacao agroforestry system can result in an accumulation of CO_2_ and dissolved inorganic carbon in the soil profile that is prone to being transported by hydrological routes to groundwater and stream water.

## Introduction

The conversion of native vegetation in production systems can result in significant emissions of CO_2_ and other greenhouse gases, especially when the changes in land use include deforestation and burning of biomass [1]. The storage of terrestrial C in soil is considered an important mechanism of the biogeochemical carbon cycle; its effect on CO_2_ emissions is considered an important potential feedback for the climate future [2–4]. The potential that soil has to represent a source or sink of CO_2_ will depend on the relation between land use, time, temperature, soil moisture, management and chemical and physical attributes of the soil [5, 6]. Several studies have reported positive correlations of the increase of CO_2_ emissions with the increase in temperature and soil moisture [7–10]. The CO_2_ resulting from enzymatic activities produced by bacteria and fungi during the decomposition that affects heterotrophic respiration of the soil may vary according to temperature, soil moisture and availability of organic matter [11,12].

The sensitivity of organic matter decomposition to temperature increases with the molecular complexity of substrate [9], suggesting litter quality could be of considerable ecophysiological importance, especially in the context of possible feedback effects of the climate [13, 14]. There are still uncertainties about the C derived from soil organic matter (SOM) sequestered in the soil: if sequestration of new forms of C following land use change exceeds decomposition (negative feedback for climatic change) or if land use change causes higher CO_2_ emissions of the soil to the atmosphere by acceleration of the decomposition of SOM [15–17].

The stocks of SOM result from the equilibrium between carbon inputs (foliar and roots detritus) and outputs (CO_2_, CH_4_ export and hydrologic runoff) [9]. The CO_2_ evasions of soil are generally estimated from measurements carried out from chambers installed in the surface [18] or modeling based on gradients of CO_2_ concentration in the soil profile [19]. However, these measurements do not correspond to total CO_2_ produced in the soil; therefore, these methods do not consider CO_2_ used in reactions of the carbonate system or carried and dissolved by the hydrologic system for underground and superficial waters [12]. This technique reflects the behavior of the main sources of CO_2_ production in soil poor in calcareous rock (root respiration and microbial decomposition of SOM). The relationship between the biological production of CO_2_ in soil with temperature and soil moisture are highlighted by models and correlations in several works [9,16,17, 19–25]. The methods for evaluating the influence of CO_2_ production processes of biological compartments in the soil profile, such as microbial decomposition, respiration of the roots, and soil abiotic factors separately still have limitations [16,25].

Tropical forests represent an important source of greenhouse gases [26]; therefore, the soil is considered an important contributor for the global balance of CO_2_ [27]. The CO_2_ production in soil is generally proportional to the amount of organic C [28] and the density of vegetation cover [29]. Thus, soil management in different crops has attracted the attention of researchers due to the potential for mitigating CO_2_ evasions [30]. The Brazilian Atlantic forest is the second largest tropical humid ecosystem of South America [31], and in relation to biodiversity, it is considered a hotspot [32]. In northeastern Brazil, part of the Atlantic forest was used for cacao plantations (*Theobroma cacao*), and the country became one of the leading producer worldwide. Much of this production (70%) consists of agroforestry systems (AFS) with perennial shade [33].

The agroforest systems of cacao substitute the understory of forests with cacao, which benefit from the shade of the canopy of the large trees [34]. Moreover, this system prevents deforestation, a process used in most production systems, and generates high amounts of organic substance in the soil due to the thick layer of typical plant litter of *Theobroma cacao* [35,36]. For this reason, some studies report that cacao AFS is a potential carbon sink [33–35]. The high C stock in the soil of this system has been widely emphasized in literature and the agroforest system of cacao has been highlighted as an alternative of production with great potential for mitigating CO_2_ emissions [35, 37–45].

Despite the understanding that soil CO_2_ emissions are related to management type and soil characteristics, there are still gaps in information about the behavior of soil in this agroforestry system. In this case, the effect of C stocks on litter and SOM on CO_2_ evasion generated in managed ASF (with shade control) and unmanaged ASF (without shading control) is poorly understood. To elucidate the dynamics of SOM and understand the behavior of cacao systems with managed and unmanaged perennial shade, CO_2_ fluxes and organic and inorganic dissolved carbon (DOC and DIC) were measured in a driest period in the profile of Ultisol, Nitosol, and Oxisol soils. In addition, the selected site was a preserved area in the Atlantic forest biome used as a “control” to establish if cacao agroforestry systems with different management affect soil C dynamics. Thus, our objectives were (I) to observe the spatial influence of litter decomposition in the first centimeters of soil for the CO_2_ flows and (II) describe the spatial variations of SOM along the profile through CO_2_ flows from different treatments and dissolved carbon concentrations in the soil solution and soil attributes. The hypothesis is that both cacao agroforestry systems (managed and unmanaged) have similar patterns of CO_2_ flows and dissolved organic and inorganic carbon compared to preserved forests.

## Material and methods

**Fig 1:**
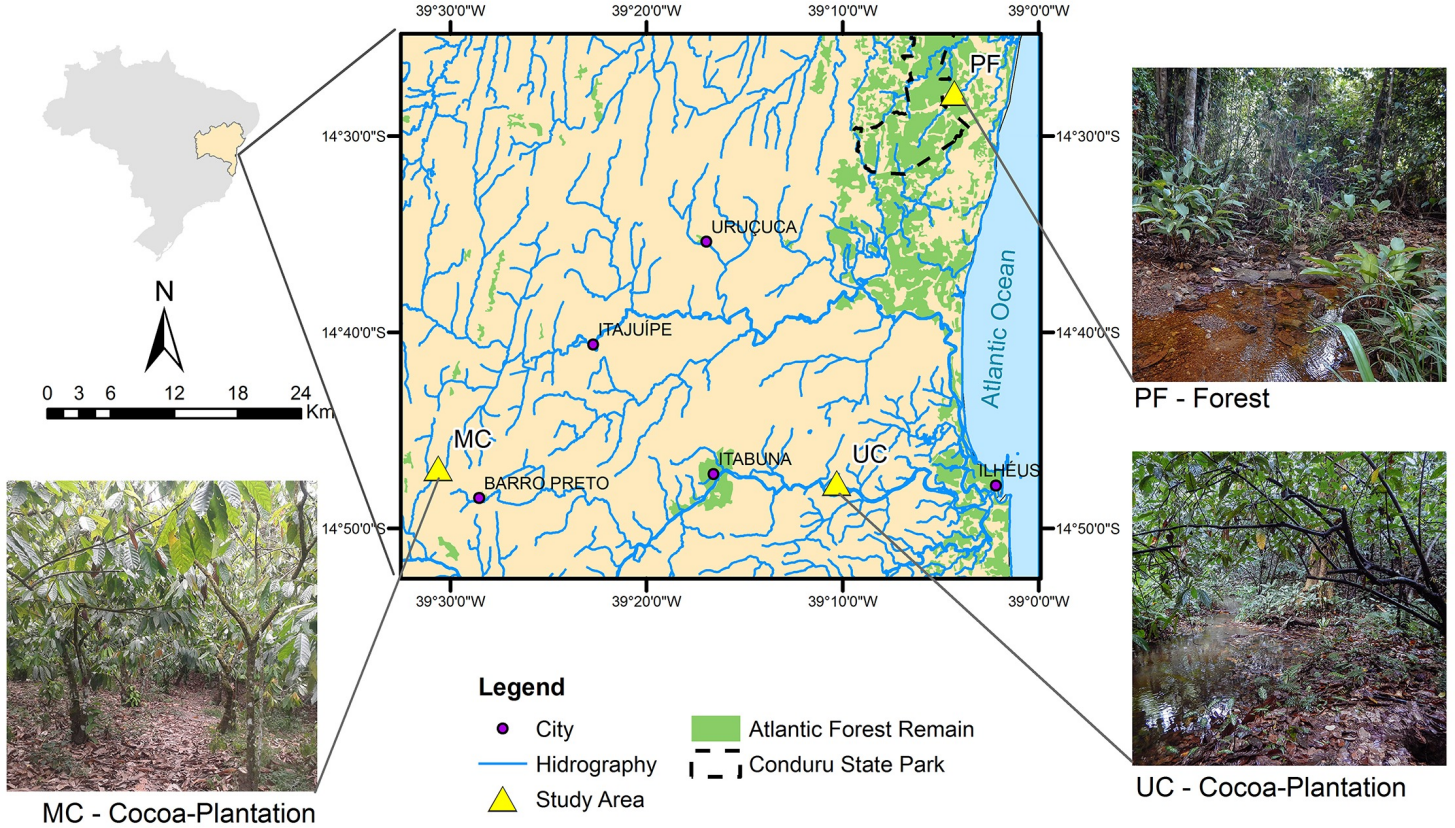
Location map of the small watersheds. (PF) Preserved forest, (MC) Managed cacao agroforestry system and (UC) Unmanaged cacao agroforestry system.

The study was conducted in three small watersheds with different soil uses (preserved forest—PF, cacao agroforestry system with management—MC and cacao agroforestry system without management—CC), located at the coordinates S142748.0 W390418.0, S144813.5 W392842.16, S144738.2 W391019.5, respectively, in northeastern Brazilian (Fig 1, Table 1). Annual average rainfall is well distributed throughout the year, with a total precipitation value ranging between 1100 and 2200 mm [45,46], and relative humidity of the air exceeds 80% [47–49]. The climate, according to the Köppen classification, is type Af. hot, humid weather, without a defined dry season.

**Table 1:** Characteristics of small watersheds.

ID	Lat/Long	Soil class	Area (ha)	Land use	Sand^b^ (%)	Clay^c^ (%)	Silt^d^ (%)
PF	S142748.0 W390418.0	Oxisol	36.08	Preserved forest	62	22	16
MC	S144813.5 W392842.16	Nitosol and Ultisol^a^	89.8	Managed cacao agroforestry system	87	10	3
UC	S144738.2 W391019.5	Ultisol	73.38	Unmanaged cacao agroforestry system	58	30	12

^a^ Soil association.

^b,c,d^ The samples for soil texture analysis were collected from 0–10 cm depths.

The study was carried out on private land, both landowners of the “Sítio Pachamama” (PF)—Divanete Souza and Veet Pramad and of the “Fazenda Nova Harmonia (MC)–Hermann Silva, gave permission to conduct the study on these site. The area located in the Universidade Estadual de Santa Cruz (UC) is released for researches of students and professors of the institution.

### Preserved forest (PF)

As described by [50,51], the soil in the area is typically petroplinthic dystropic latosol (Oxisol) with a sandy texture at a proportion of 62% of sand, 16% of silt and 22% (Table 1) clay and pH 4.5. The vegetation consists of a conglomerate of several regeneration stages [52]. It is classified as a *Submontane Ombrophilous* dense forest that boasts a uniform canopy over 25 m tall with a few emerging individuals, many epiphytes, large vines, and a dense understory [53].

### Managed cacao agroforestry system (MC)

The soil of the area is associated with saprolyte eutroferic haplic nitosol, red-yellow abrupt eutrophic argisol (Ultisol), rich in nutrients, with a great potential for agricultural production [47]. The soil has a sandy texture with 81% sand, 16% clay and 3% silt (Table 1). This system has an extensive vegetation cover due to the cacao plantation (34.1 ha), with a forest patch in its central portion and two areas in regeneration process (4.5 ha) [47]. The main management strategy is shade control and it is represented by a proportion of 24% of native trees and 76% of cacao trees around 150 years old (according to the inventory of the property). Periodically, the cacao trees are pruned and all shrubby vegetation is removed; however, this organic matter is deposited in the soil.

### Unmanaged cacao agroforestry system (UC)

As described by [54], the soil of the area is classified dystrophic argisol (Ultisol) as typical A to moderate eutrophic soil with medium clayey texture, with the proportion of 58% sand, 12% silt, and 30% clay (Table 1) and pH 4.3[50]. The site is located on a slope of roughly 5%, and for 20 years, there has been no record of a typically managed cacao agroforestry system procedure. Despite the initial current shading with 30% of native trees and 70% of cacao trees [55], the absence of shading control or bush pruning has led the native vegetation to settle similarly to a natural forest.

### Data collection and sampling

**Fig 2:**
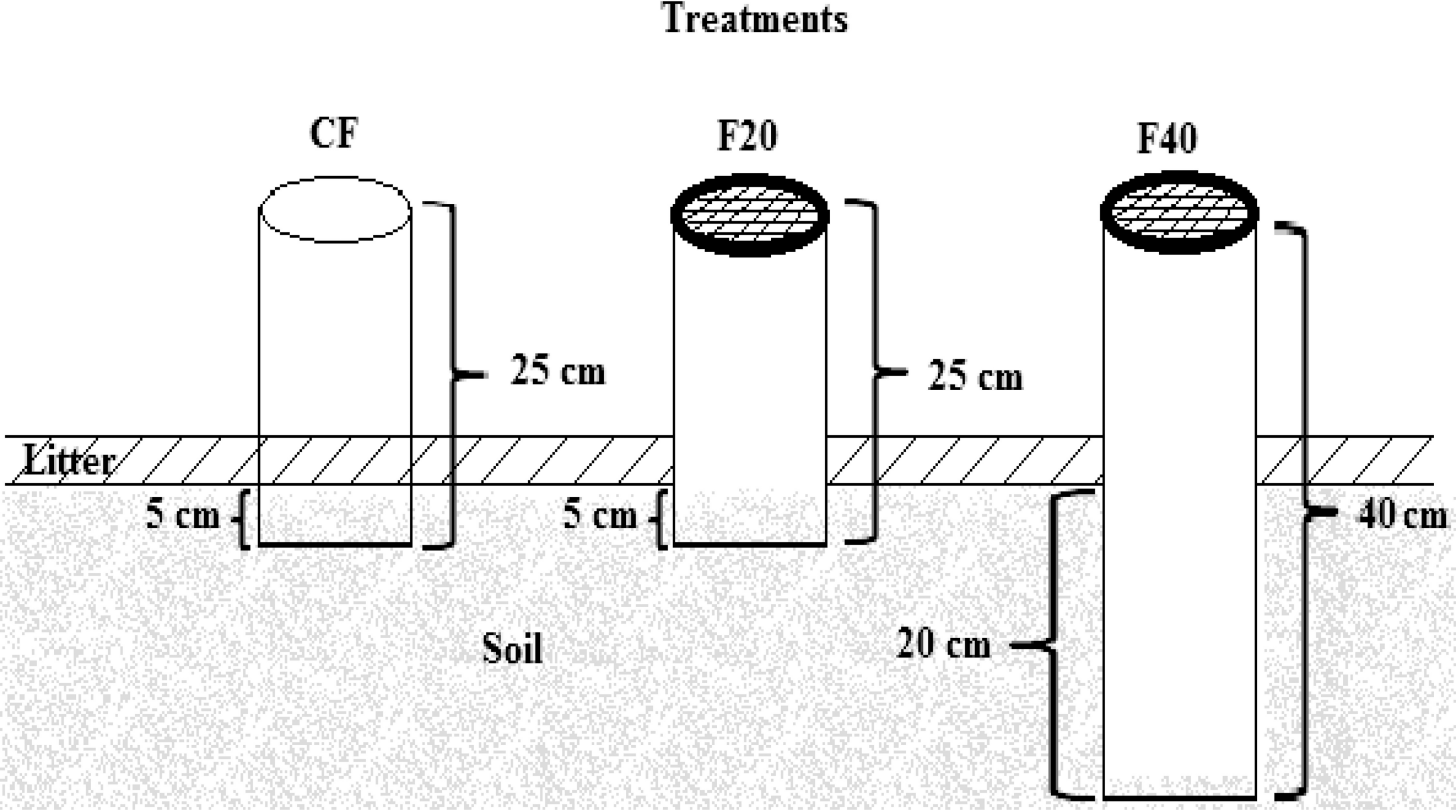
Treatments for CO_2_ measurements. (CF) Controls flux, (F20) Flux of 20 cm, (F40) Flux of 40 cm.

Sampling occurred every two weeks for six months of the dry period, from December 2015 to May 2016. Data on rainfall events from 1 December 2015 to 30 November 2016 were obtained from the Climate Monitoring Program in Real Time in the Northeast Region (Proclima). Soil gas was monitored with PVC fixed chambers, distributed according to the conditions of the environment in a plot within an area of 25 x 25 m. In each area, a total of 10 rings made of PVC with 15 cm in diameter were installed with three types of treatment (Fig 2): 2 controls (25 cm high, set with plant litter, fixed with 5 cm penetration in the soil), 4 rings of 20 cm (25 cm high with fixed height, litter removed within the ring, fixed with 5 cm penetration in the soil) and 4 rings of 40 cm (fixed in trenches 20 cm deep). To avoid disturbances and accumulation of plant litter inside the rings, with the exception of the control, the 25 and 40 cm rings remained covered with nylon mesh, removed only for measurements.

The soil samples were collected at depths of 0–20, 20–40, 40–60, 60–80 and 80–100 cm. For each depth, composite samples were collected from three different points, distributed according to the viability of the environmental conditions. Soil analyses were carried out in the Laboratory of Soil Science at ESALQ/USP according to the manual of chemical analysis to assess the fertility of tropical soils (IAC, 2001). The pH was determined in CaCl_2_ 0.01 mol L^-¹^; phosphorus (P) was determined using the colorimetric method extracted with ion exchange resin; potassium (K) was determined by extraction with ion exchange resin and determined using atomic emission spectrophotometer; calcium (Ca) and magnesium (Mg) were determined by extraction with ion exchange resin and determined in an atomic absorption spectrophotometer. The potential acidity extraction was analyzed using SMP buffer.

#### Temperature and soil moisture

For each collection event, parameters such as temperature and soil moisture next to the chambers were recorded at two depths: 0–10 cm and 0–20 cm. For the temperature, a mobile digital thermometer, the same used to measure the air temperature, was inserted 5 cm into the soil. Soil samples were collected, packed in plastic bags and weighed on the same day of collection. For the soil moisture measurement, 10 g of soil were weighed (w1) and dried in an oven at 55°C until reaching constant weight (w2). Soil moisture (SM) expressed in % was determined using Eq 1, where w1 and w2 were expressed in kg.

$$SM=100\frac{\left(w1-w2\right)}{w2}$$ (1)

#### Measures of CO_2_ fluxes from soil surface

The measurements were initiated 15 days after the rings were installed, generally taken from 09:00 h to 12:00 h in the morning, and each chamber was measured for 10 min. The PVC cover 4.5 cm high was coupled to an infrared gas analyzer (Li-Cor 820) and a scrubber containing drierite to remove moisture from the air. The cover had a vent to allow equalization of chamber pressure with atmospheric pressure. Air was circulated between the chamber and the Li-Cor by a pump with a flow of air 1 L^-1^ min ^-1^ through the “Bev-a-line Tubing”. CO_2_ concentrations (ppm) within the chamber were recorded up to 5 minutes after placing the cover on the ring. Before each incubation, atmospheric CO_2_ was measured for 5 min and the reading was recorded in real time by a datalogger at an interval of 5 seconds. The Li-Cor was frequently calibrated in standard mixture gas at the laboratory.

CO_2_ fluxes were calculated from linear regression by the dynamic chamber method, where headspace gas concentration changes over time (Eq 2): where F = CO_2_ flow in mg CO_2_—C m^2^ h^-1^; V = the internal volume of the chamber in L; A = area of the chamber in m^2^; dc/dt = slope of the change in CO_2_ concentration as a function of time in μmol L^-1^; t = time (t = 0).

$$F=\left(\frac{dc}{dt}\right).\left(\frac{V}{A}\right).t$$ (2)

#### CO_2_ from litter decomposition

The influence of the litter decomposition compartment (LD) in total CO_2_ emissions in land/atmosphere interface was calculated subtracting the 20 cm flux (representing CO_2_ flow from soil without the litter layer) from total CO_2_ flow represented by control treatment (autotrophic respiration from plant roots plus heterotrophic respiration from soil organisms) as shown in Eq 3:

LitterDecomposition=ControlsFlux−Fluxof20cm (3)

#### Organic carbon and dissolved inorganic carbon (DOC and DIC) in soil solution

In total, six individual tension lysimeters were installed. In each small watershed, three extractors were installed, one for each depth (15, 45, and 90 cm). The soil solution (SS) was extracted from the extractor using a 60-ml syringe and hose. Manual pressure (vacuum) was applied with a syringe and needle prior to extraction. After lysimeters were installed and equilibrated for a 15-day period, the first samples were discarded. All samples from each collector were treated on site by filtration and 60 ml was filtered through a glass microfiber membrane (pore: 0.7 μm, combusted at 450°C for 4 h), transferred to combusted glass bottles and preserved using mercuric chloride (HgCl_2_) until analysis. DOC and DIC were determined using a total organic carbon analyzer with infrared ray detector (Shimadzu model TOC-V_CPN_).

#### Statistical analysis

The non-parametric Mann-Whitney test with p<0.05 was used to compare the averages of the CO_2_ fluxes and dissolved C between the areas. The multivariate analysis of the main principal components (PCA) was used to test the temporal interaction between precipitation, temperature, soil moisture and the fluxes of CO_2_ (from F20 and F40 treatments). All analyses were performed with PAST 1.91 [56]. All data are within the paper and can be found as a Supporting Information files (S1–S4 Tables).

## Results

**Fig 3:**
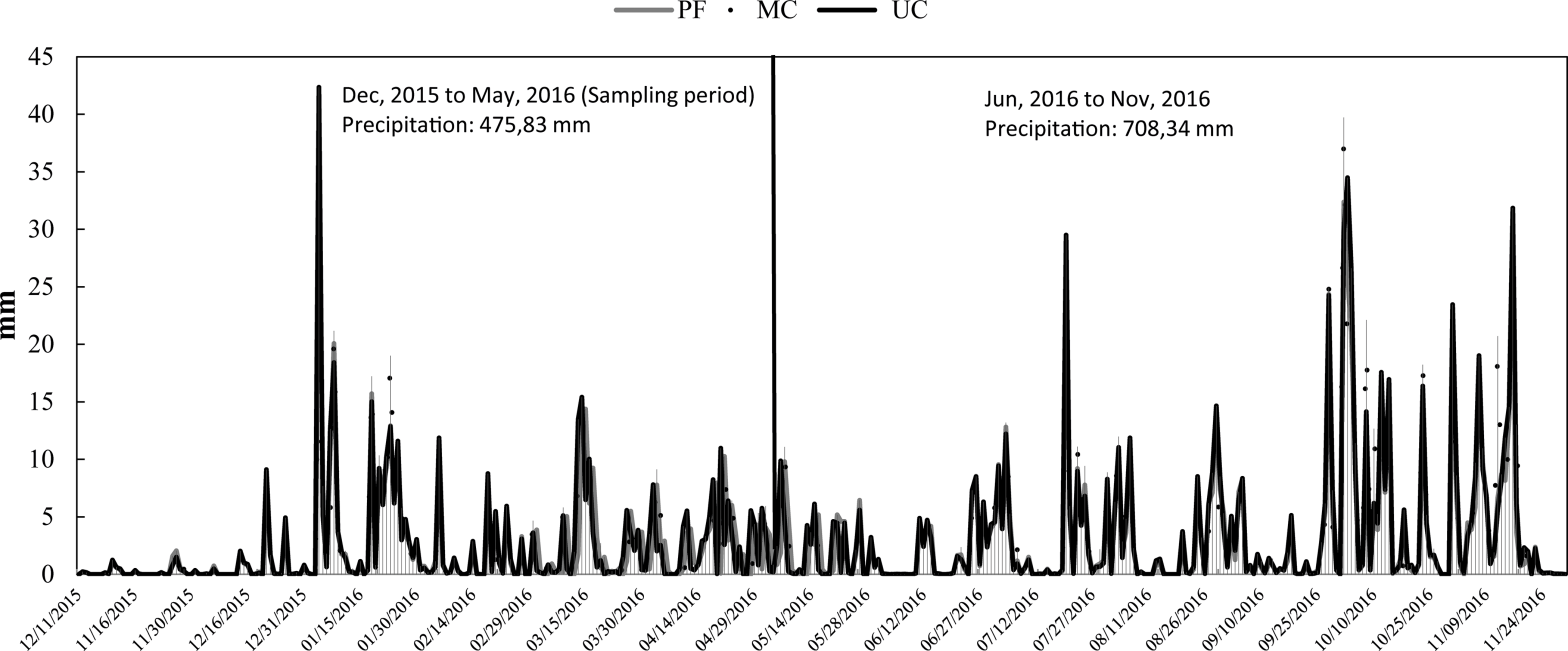
Daily rainfall of the small watersheds. (PF) Preserved forest, (MC) Managed cacao agroforestry system and (UC) Unmanaged cacao agroforestry system.

Throughout the sampling period, a similar rainfall distribution was observed in the three areas. The maximum values were recorded in the first few weeks of January and in the second and third weeks of March. A rainfall value of around 1 mm was recorded in most of the sample periods and in December 2015 minimum values of 0.1 mm were observed in three areas (Fig 3).

**Fig 4:**
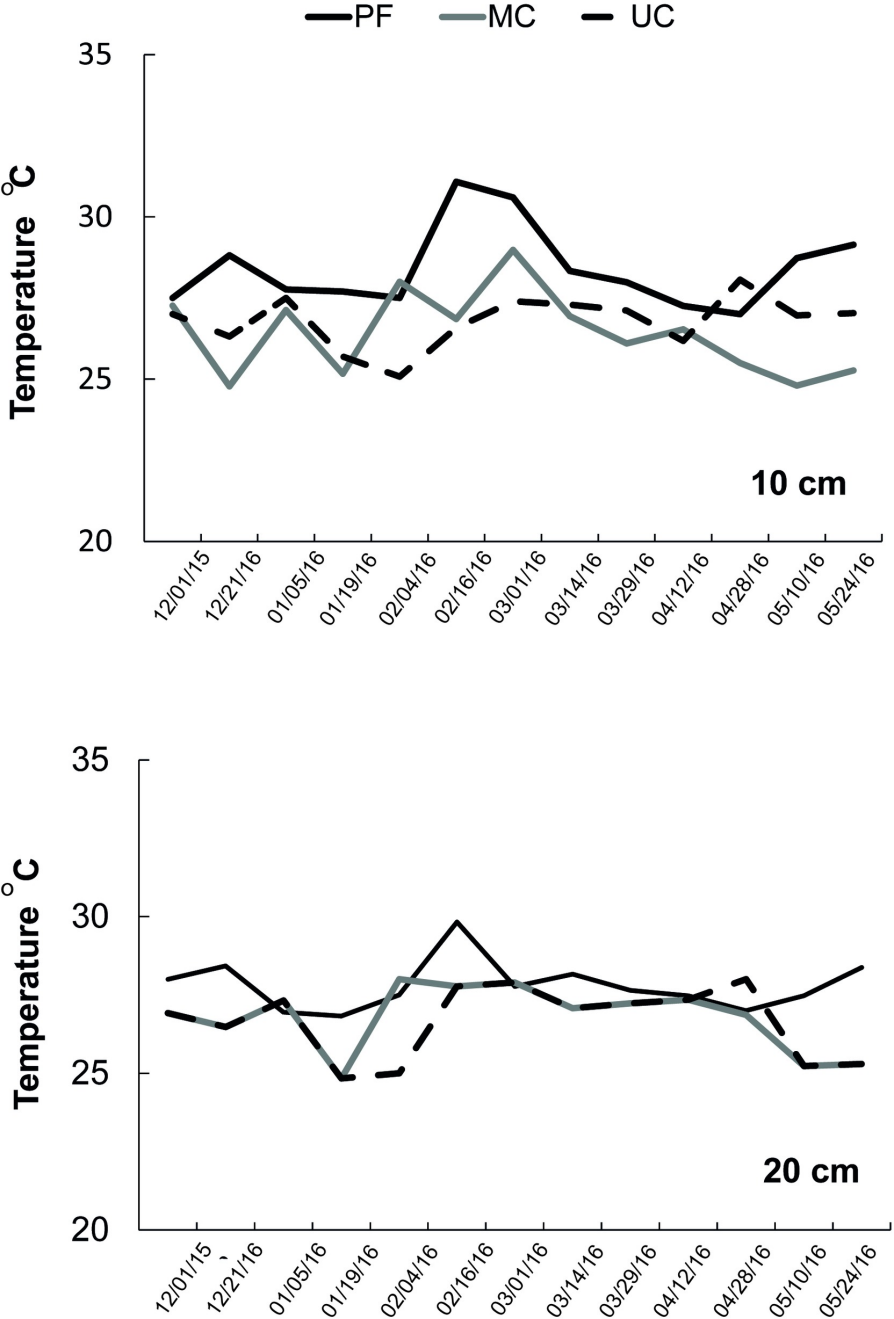
Soil temperature (°C) of the small watersheds. (A) 10 cm depth to Preserved forest—PF *n =* 53, Managed cacao agroforestry system- MC *n* = 55, Unmanaged cacao agroforestry system—UC n **=** 57 and (B) 20 cm depth for Preserved forest—PF *n =* 40, Managed cacao agroforestry system—MC *n* = 41, Unmanaged cacao agroforestry system—UC n **=** 39.

Soil temperatures at depths of 10 and 20 cm did not show great variation between the three areas, with values around 27.3° C (Fig 4, S1 Table). In the three areas, the highest temperatures were registered between February and March with 30.7°C in PF and MC, and 27° C in UC. The minimum temperature was 25.8°C, with steadier values in UC compared to PF and MC (Fig 4,).

**Fig 5:**
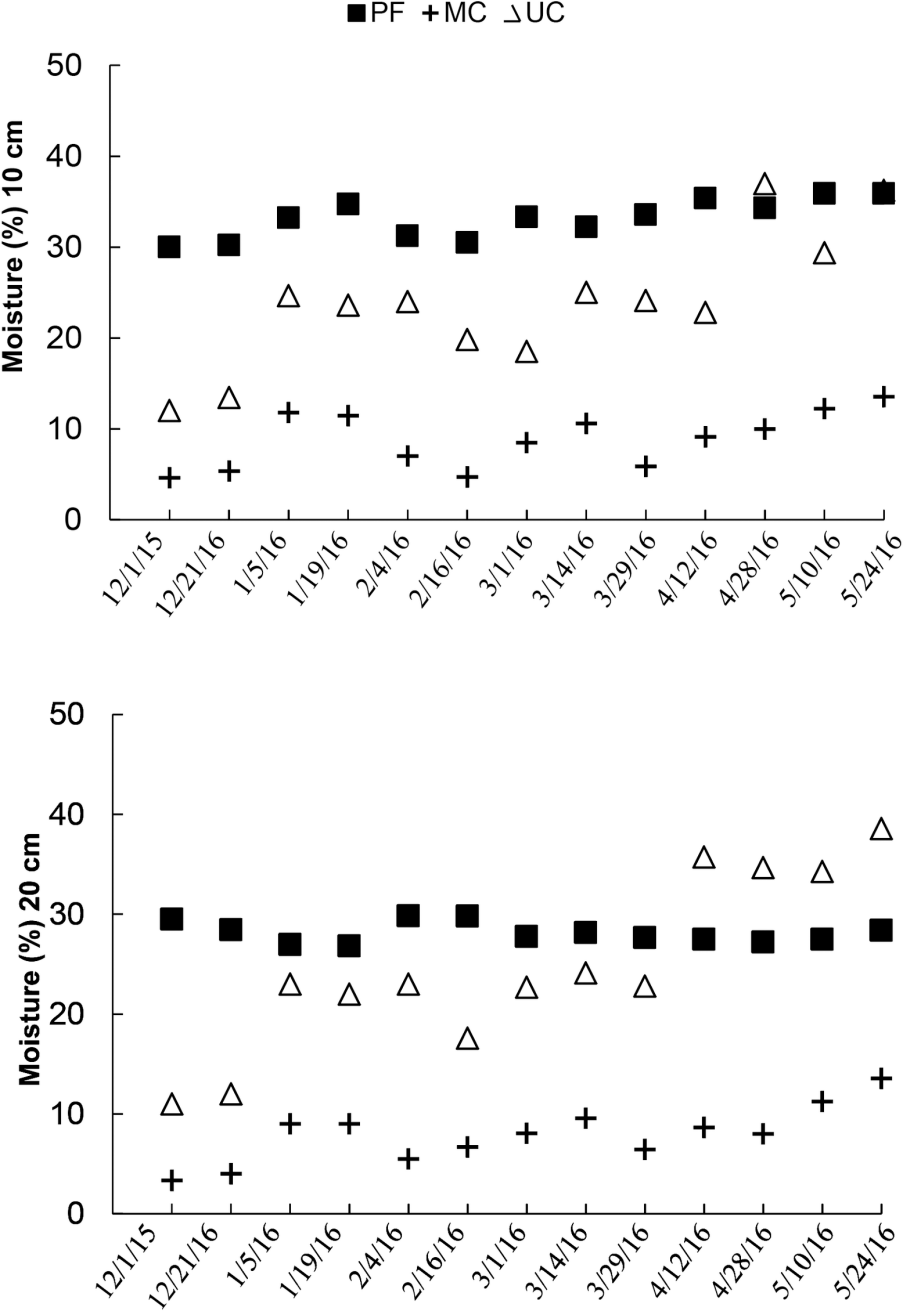
Soil moisture (%) of the small watersheds. (A) 10 cm depth to Preserved forest—PF *n =* 32, Managed cacao agroforestry system- MC *n* = 33, Unmanaged cacao agroforestry system—UC n = 42 and (B) 20 cm depth for Preserved forest—PF *n =* 28, Managed cacao agroforestry system—MC *n* = 26, Unmanaged cacao agroforestry system—UC n **=** 30.

Soil moisture was different between the areas but it did not vary between the depths (Fig 5, S2 Table). The highest values were recorded in PF, with 36.8 and 38.6%, and the minimum was recorded in MC, with 4.6% and 4.1%, for 10 and 20 cm, respectively. In UC, the minimum values were recorded in the less rainy week in December 2015 with 13.5% in both depths, and the maximum of 32.8% in 10 cm and 34.5% in 20 cm were observed in May (Fig 5).

Although the chemical characteristics found in the three areas demonstrate soils with distinct conditions (Table 2), the values of variables such as pH, K^+^ and P were found in the same range of values in PF, MC and UC. In this case, the pH was acidic in all soil profiles, varying from 4.1 to 4.6, and K^+^ concentrations were around 0.9 mmolc dm^-3^. Concerning P, the values varied from 4 to 14 mg dm^-3^ and neither had a pattern of distribution correlating with concentrations at the different depths (Table 2). In all the areas, OM concentrations decreased as the depth increased. In PF and MC at the depth of 80–100 cm, SOM concentrations were almost 3 times higher than UC. However, in the first centimeters of the soil (from 0–20 cm), SOM in PF and UC were similar (31 and 25.5 g dm^-3^, respectively), whereas SOM in MC was about 50% less (16 g dm^-3^). Fe and H+Al concentrations were lower in PF, with different orders of magnitude, compared to MC and UC, where the concentrations tended to increase with depth (Table 2). The same trend was found in the values of the sum of exchangeable bases (SB). They were higher in MC and UC, compared the PF; and in this case, they did not present a pattern in the distribution of the concentrations.

**Table 2:** Soil chemical characteristics.

PF											
Depth (cm)	pH	OM (g dm ^-3^)	P (mg dm^-3^)	Fe (mg kg ^-1^)	K^+^	Ca^+2^	Mg^+2^	H+Al	BS	CEC	V (%)
					(mmolc dm-^3^)						
0–20	4.3	31	9.5	30.6	<0.9	6	2.5	4.3	9.3	40.8	23
20–40	4.5	15.5	7	19.8	<0.9	2.5	1	1.7	3.9	30.5	13
40–60	4.3	10.5	7	13.4	<0.9	4.5	1	2.7	6	32.5	18.5
60–80	4.6	10	5.5	13.4	<0.9	2.5	0.9	1.7	3.4	24.9	13.5
80–100	4.5	9	5.4	27.4	<0.9	2.5	1	1.7	3.8	25.8	15
MC											
0–20	4.2	16	10	75.6	1.05	10.5	5.5	21.5	17.1	38.5	31.1
20–40	4.2	9.5	5	34.1	<0.9	6	2	37	6.5	45.5	9
40–60	4.2	4	4	92.3	<0.9	6	2.5	36.5	9.2	45.6	10
60–80	4.1	3.5	4.5	31.9	0.95	6	4	34	10.8	39.8	17.5
80–100	4.5	11	14	166.6	1.1	14	6	12	21.1	21.1	33.1
UC											
0–20	4.5	26.5	10.5	109.2	1	19	9.5	25.5	39.5	55.1	54
20–40	4.1	10	5	61.5	<0.9	6	3	23.5	20.6	33.2	29
40–60	4.3	10.5	4.5	184.1	<0.9	9	5.5	17	23.1	32.1	47
60–80	4.5	6.5	4.5	177.8	<0.9	10.5	6	15.5	17.3	32.7	53
80–100	4.5	3.5	10	219.8	1	10	6.5	14.5	17.2	31.6	53.5

PF, preserved forest; MC, managed cacao agroforestry system; UC, unmanaged cacao agroforestry system; Depth, soil profile; OM, organic matter; P, phosphorus; Fe, iron; K^+^, potassium; Ca^+2^, calcium; Mg^+2^, magnesium; H+Al, potential acidity; SB, sum of exchangeable bases; CEC, cationic exchange capacity; V, saturation of CEC by bases.

In general, concentrations Ca^+^ and Mg^+^ ions were also higher in the two agroforestry systems MC and UC (Table 2). The values were higher mainly at depths of 0–20 (MC with 10.5 and 5.5 mmolc dm^-3^ and UC with 19 and 9.5 mmolc dm^-3^ of Ca^+^ and Mg^+^, respectively) and at the depths of 80–100 cm (MC with 14 and 6 mmolc dm^-3^ and UC with 10 and 6.5 mmolc dm^-3^ of Ca^2+^ and Mg^2+^, respectively). In PF, the concentrations increased at depths of 0–20 cm with 6 and 2.5 mmolc dm^-3^ of Ca^2+^ and Mg^2+,^ respectively.

**Fig 6:**
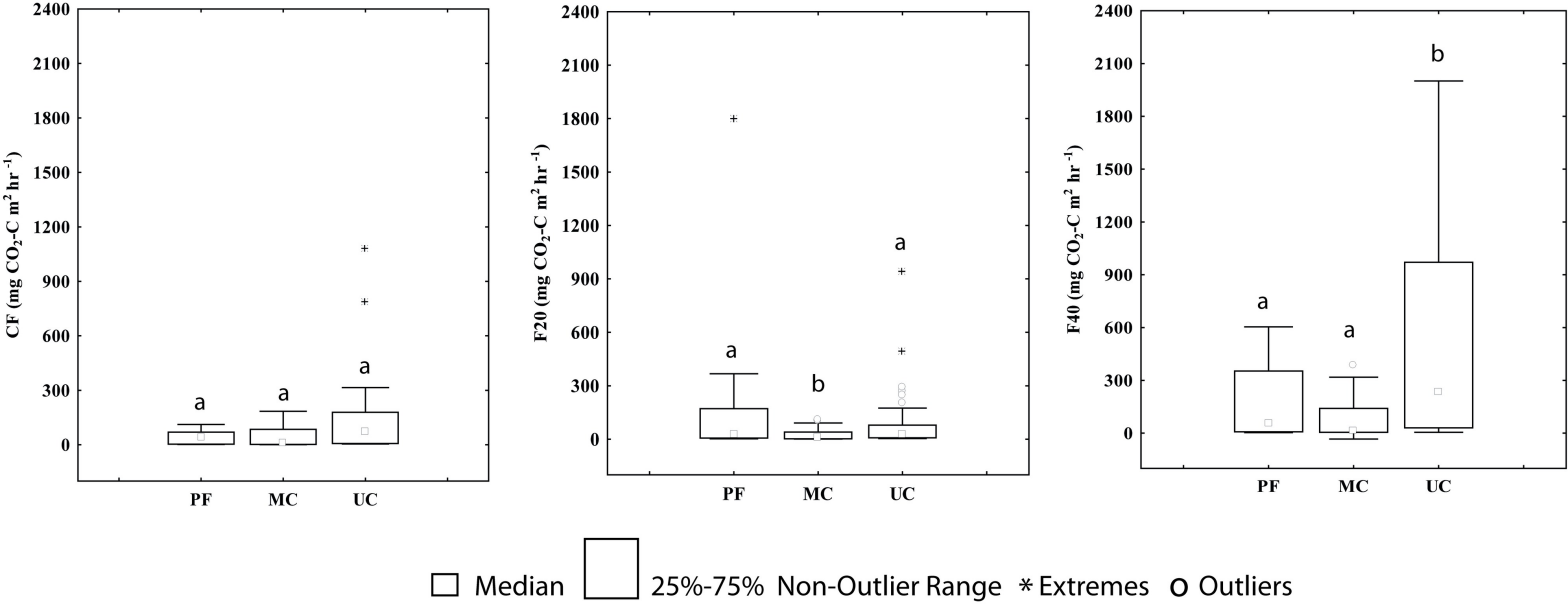
Spatial variation of the CO_2_ fluxes (mg CO_2_—C m^2^ h^-1^). (CF) Control fluxes, (F20) chambers with 20 cm and (F40) chambers with 40 cm, (PF) Preserved forest, (MC) Managed cacao agroforestry system and (UC) Unmanaged cacao agroforestry system. Different letters indicate different values for the statistical Mann-Whitney *U*-test, with a *p* < 0.05.

The CO_2_ flows in the control treatment (CF) were higher in UC (125 mg CO2—C m^2^ h^-1^) than MC and PF, where the CO_2_ fluxes were similar (41.8 and 45.03 mg CO2—C m^2^ h^-1^, respectively); however, the values did not differ significantly between the three areas (*p*<0.05) (Fig 6, S3 Table). The CO_2_ flows of the 20 cm treatments (F20) from PF were higher than in UC (79.5 and 65.3 mg CO2—C m^2^ h^-1^, respectively), and both differed statistically (p<0.05) from MC, which had the lowest flows (24.3 mg CO2—C m^2^ h^-1^)

Among the three types of treatments used (control, F20 and F40), F40 recorded the highest CO_2_ fluxes in the three areas, especially in UC with an average value of 601.1 mg CO2—C m^2^ h^-1^ (Fig 6). This average was four times higher than PF (163.3 mg CO2—C m^2^ h^-1^) and eight times higher than MC (82.7 mg CO2—C m^2^ h^-1^). In this case, the CO_2_ of F40 in UC was significantly different (*p*<0.05) in relation to PF and MC, which did not differ (Fig 6).

**Fig 7:**
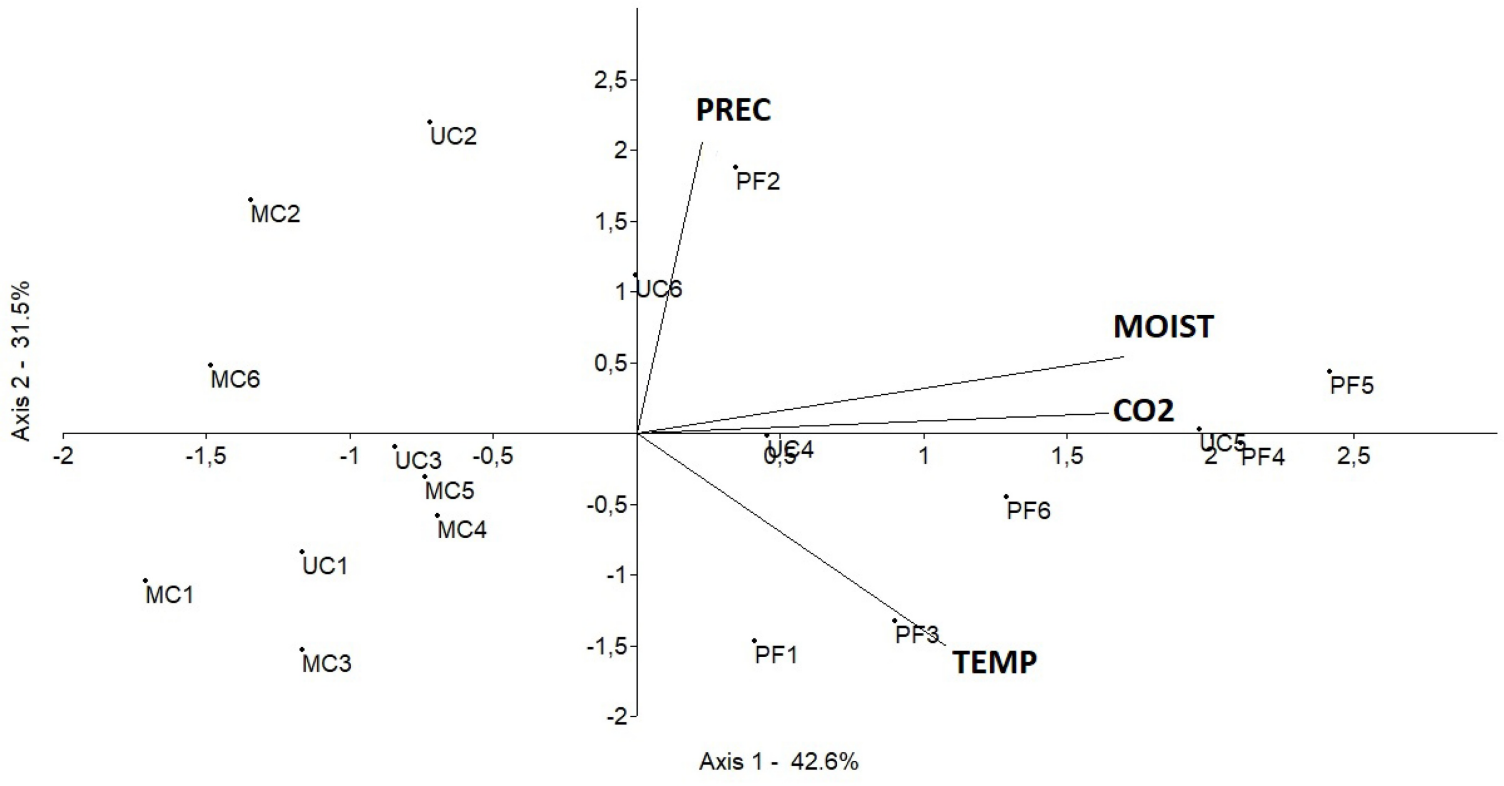
Principal component analysis (PCA) in monthly timescale determined by Axis 1 and Axis 2. (CO_2_) CO_2_ fluxes from the 20 cm treatment in mg CO2—C m^2^ h^-1^, (PREC) Precipitation in mm, (TEMP) temperature in °C, (MOISTURE) Soil moisture in %, (PF) Preserved forest, (MC) Managed cacao agroforestry system and (UC) Unmanaged cacao agroforestry system, (1, 2, 3, 4, 5 and 6) December, January, February, March, April and May, respectively.

**Fig 8:**
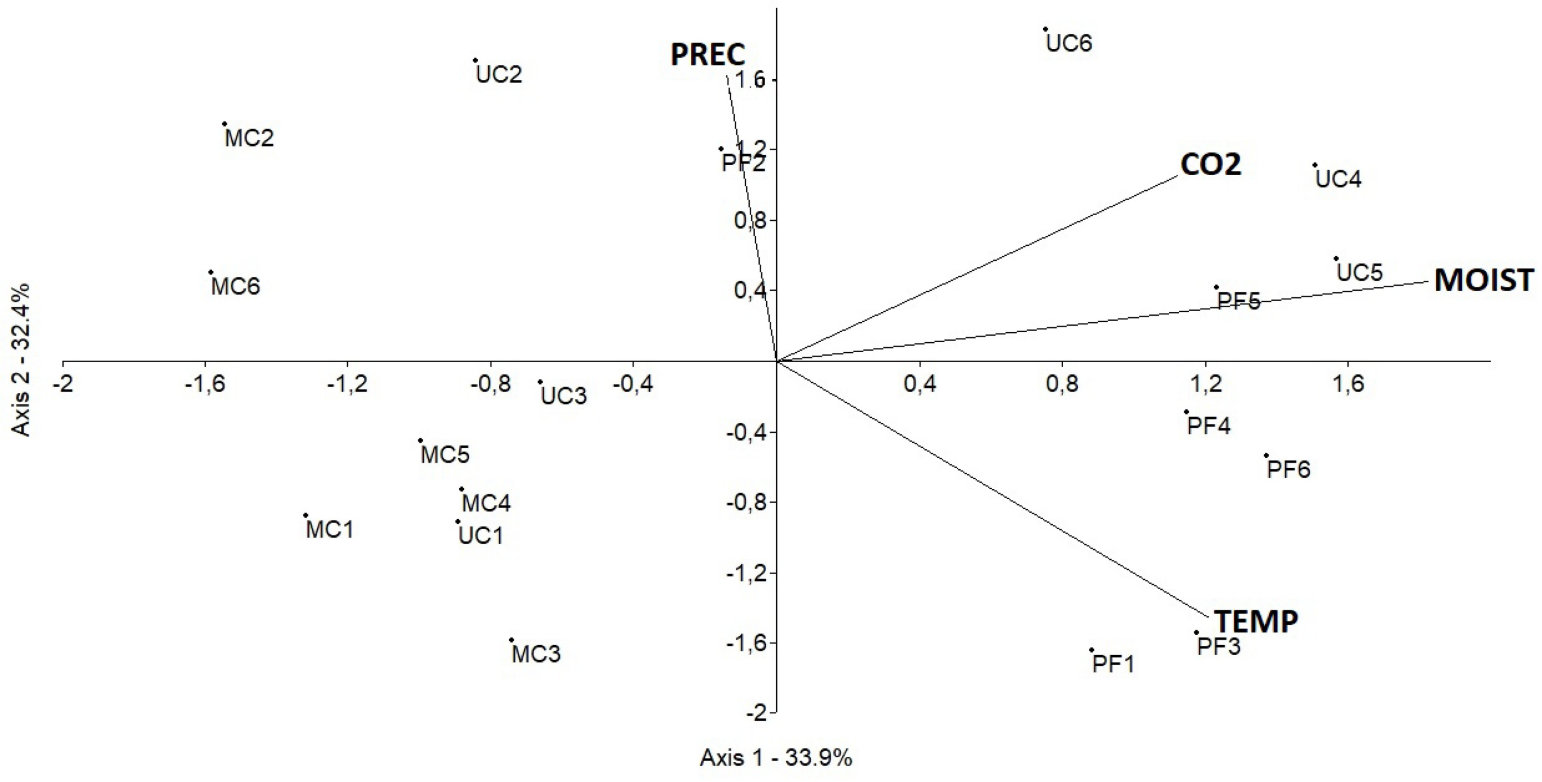
Principal component analysis (PCA) in monthly timescale determined by Axis 1 and Axis 2. (CO_2_) CO_2_ fluxes from the 40 cm treatment in mg CO2—C m^2^ h^-1^, (PREC) Precipitation in mm, (TEMP) temperature in °C, (MOISTURE) Soil moisture in %, (PF) Preserved forest, (MC) Managed cacao agroforestry system and (UC) Unmanaged cacao agroforestry system, (1, 2, 3, 4, 5 and 6) December, January, February, March, April and May, respectively.

PCA analysis showed that in the 20 cm and 40 cm treatments, precipitation, temperature and soil moisture influence the CO_2_ fluxes only in PF and UC (Figs 7 and 8).

In the 20 cm treatment, the CO_2_ fluxes were arranged on the positive side of the main axes and directly related with precipitation in January in PF (PF2) and in May in UC (UC) (74.13% of the total variance). Also, soil moisture was directly related to CO_2_ fluxes in April, March and May in PF and UC (PF4, PF5, UC4, UC5 and U6). In December and February, temperature inversely related to the CO_2_ fluxes in PF (PF1 and PF3).

In the 40 cm treatment, PCA analysis showed that the CO_2_ fluxes were arranged on the positive side of the main axes (66.34% of total variance), and that in March and April in PF and UC (UC4, PF5, UC5), they directly related to soil moisture (Fig 8). In PF temperature in December and February (PF1 and PF3, respectively) and precipitation in January inversely related to the CO_2_ fluxes (Fig 8).

**Fig 9:**
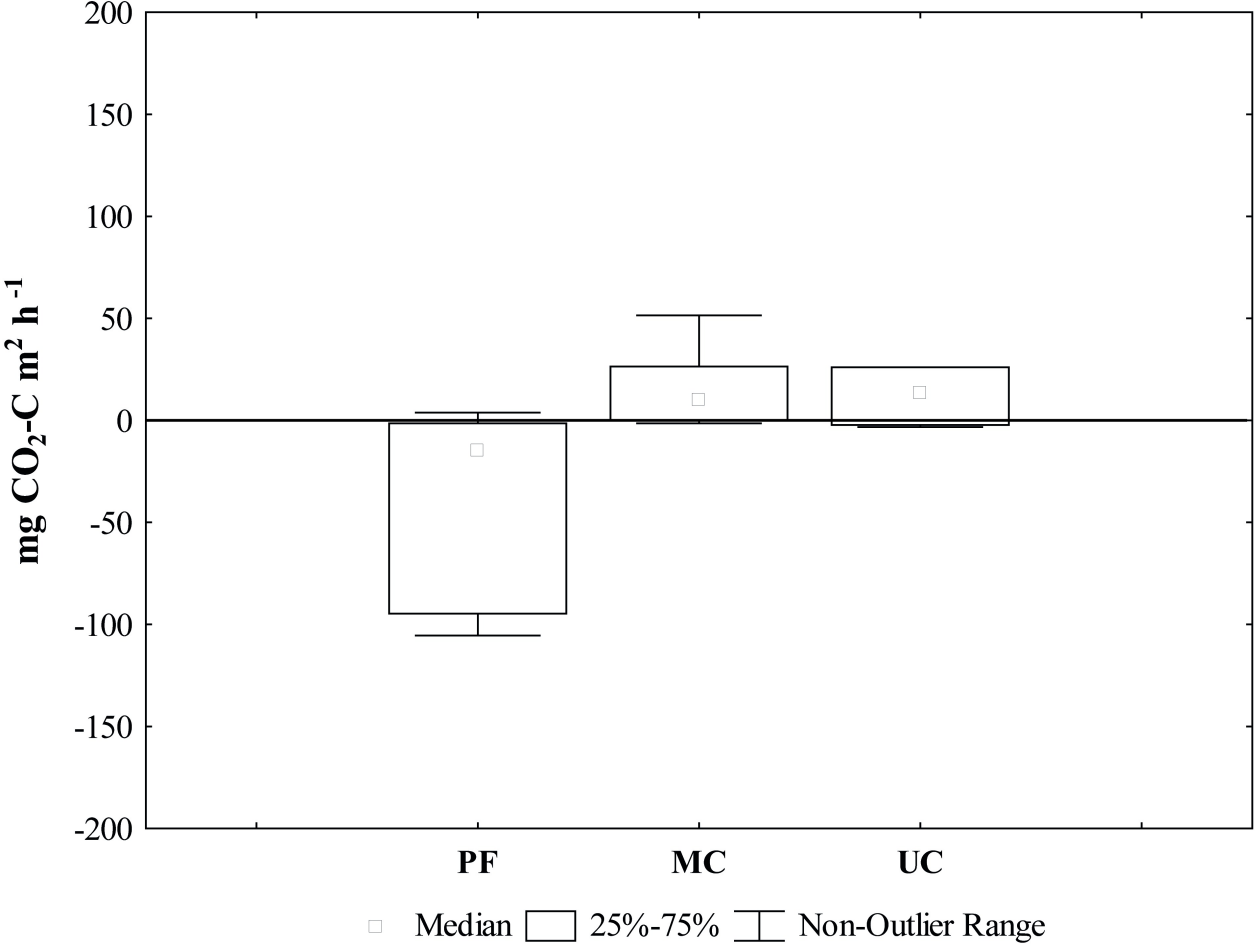
Spatial variation of CO_2_ fluxes from litter layer decomposition (mg CO_2_—C m^2^ h^-1^). (PF) Preserved forest, (MC) Managed cacao agroforestry system and (UC) Unmanaged cacao agroforestry system.

In MC and UC, CO_2_ production in the litter layer from litter decomposition (LD), calculated as the difference between the measured control flux (CF) minus the F20 flux, was 10.6 and 13.4 mg CO2—C m^2^ h^-1^, respectively (Fig 9). In PF, however, the LD flux was negative (-14.6 mg CO2—C m^2^ h^-1^), indicating the disturbance from removing the litter layer in the F20 chambers or spatial heterogeneity may have caused an artifact (Fig 9).

**Fig 10:**
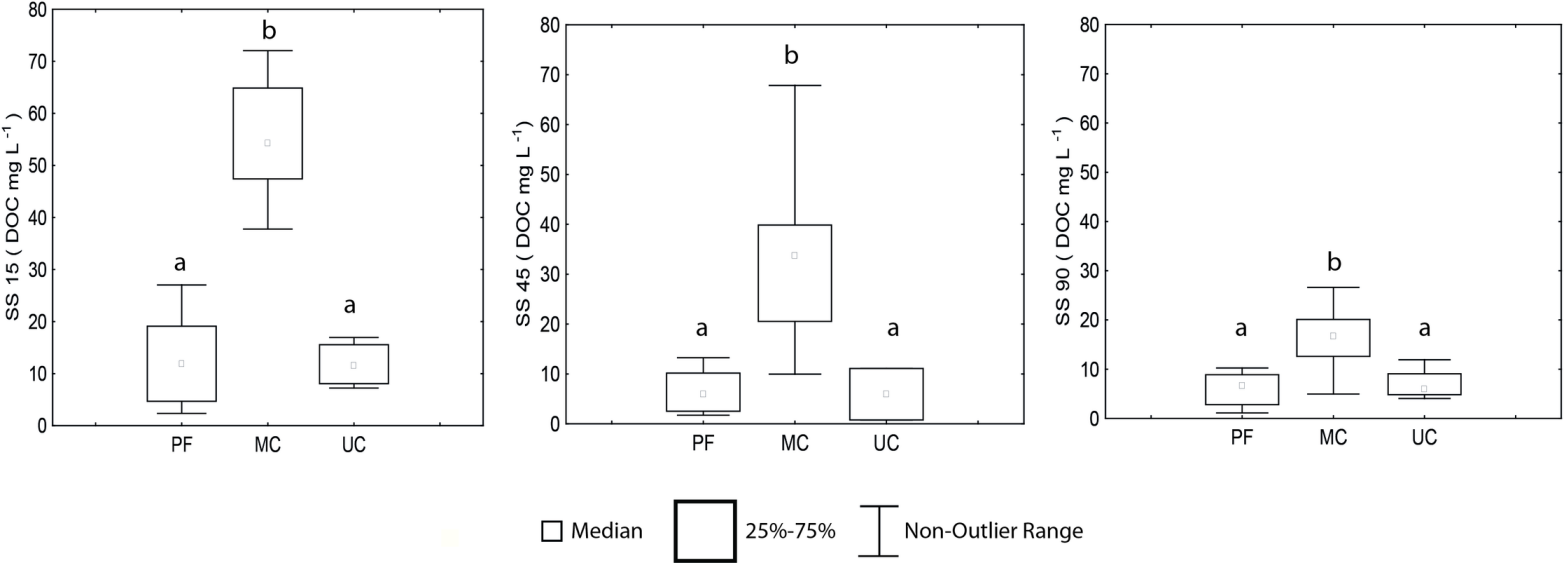
Spatial variation of dissolved organic carbon–DOC–(mg L^-1^) in soil solution (SS) at depths 15 cm (SS15), 45 cm (SS45) and 90 cm (SS90). (PF) Preserved forest n = 8, 16 and 13, (MC) Managed cacao agroforestry system n = 6, 9 and 9 and (UC) Unmanaged cacao agroforestry system n = 5, 5, 5 to SS15, SS45 and SS90 respectively. Different letters indicate different values for the statistical Mann-Whitney *U*-test, with *p* < 0.05.

In the soil profile, the DOC concentrations decreased with increasing depth (Fig 10, S4 Table). In MC, the highest recorded concentrations of DOC were recorded in soil solutions SS15, SS40 and SS90 (55.2, 32.2 and 16. 2 mg L ^-1^, respectively), compared to PF (12.6, 6.7 and 8.8 mg L ^-1^, respectively) and UC (11.8, 5.9 and 6.9 mg L ^-1^, respectively). Thus, the three depths in MC had statistical differences compared to PF and UC (*p*<0.05) and there were no statistical differences between PF and UC (*p*<0.05) (Fig 10).

**Fig 11:**
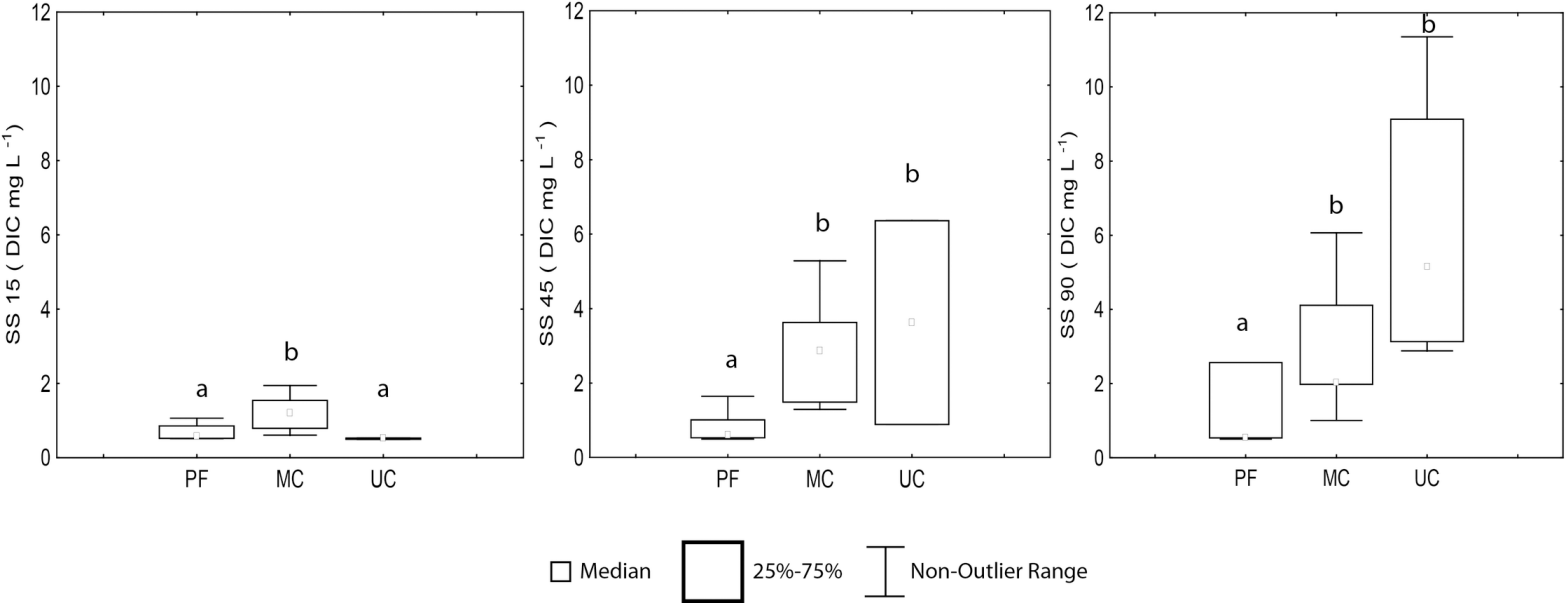
Spatial variation of dissolved inorganic carbon–DIC–(mg L^-1^) in soil solution (SS) at depths 15 cm (SS15), 45 cm (SS45) and 90 cm (SS90). (PF) Preserved forest n = 8, 16 and 13, (MC) Managed cacao agroforestry system n = 6, 9 and 9 and (UC) Unmanaged cacao agroforestry system n = 5, 5, 5 for SS15, SS45 and SS90 respectively. Different letters indicate different values for the statistical Mann-Whitney *U*-test, with *p* < 0.05.

In the soil profile, with the exception of PF where DIC distribution was relatively uniform, the concentrations increased with depth (Fig 11, S4 Table). Compared to SS15 and SS45, the highest concentrations were registered in SS90 with 3.6 and 6.1 mg L ^-1^ (for MC and UC, respectively) (Fig 11). In UC, the DIC concentrations in SS90 were up to four times higher than PF (0.5 mg L ^-1^), with statistical differences between them (*p*<0,05) and no statistical difference with MC.

## Discussion

Land use influences the production and consumption of CO_2_ according to the vegetation cover [57, 25]. In this study, although the highest total of CO_2_ fluxes were found in managed and unmanaged cacao agroforestry systems (MC and UC, respectively), these values were not statistically different from those measured in the preserved forest (PF).

The total CO_2_ flux considered in this work was represented by the control treatment that includes sources of CO_2_ from the autotrophic respiration of plant roots and heterotrophic respiration from soil organisms. Few works have made these distinctions, mainly due to difficulties isolating these CO_2_ sources [16,58]. In addition, although our study included measurements of CO_2_ emissions between 9:00 am and 12:00 am, it is important to note that fluctuation of CO_2_ emissions occur all day. This fluctuation was reported by [59] when comparing the diurnal fluctuation of CO_2_ between a grass monoculture without live fence and a grass culture with live fence. This study showed the lowest CO_2_ emissions were reported between 6:00 am and 12:00 am and the highest between 12:00 pm and 6:00 pm. Also, the trees of the live fence reportedly contributed to minimizing CO_2_ fluctuation during daylight hours. Therefore, considering the cacao agroforestry systems and the preserved forest have a full canopy, it is suggested that, as in the case of the live fence, it can contribute to the mitigation of CO_2_ emissions fluctuations throughout the day.

Several studies have demonstrated that CO_2_ fluctuations are related to temperature conditions, rainfall events, soil moisture, chemical and biological factors [19,20,22,24,58–62]. Our study was carried out in an unusual year with low annual precipitation totalizing 1183.37 mm. From December 2015 to May 2016, precipitation was only 475.83 mm with longer periods of drying followed by rapid rewetting (Fig 3). This suggests that the Birch effect may have occurred in all three study areas (PF, MC and CC). Therefore, the Birch effect with the other biotic and abiotic factors could explain the high standard deviation observed in the distribution of CO_2_ fluxes in all treatments in the three studied areas. The wetting pulse may be attributed to the mineralization of previously unavailable, easily decomposable organic substrates, possibly causing an extra short-term boost in microbial activity for a few days that exceeds the microbial activity of a permanently moist soil [63,64].

The influence of variables such as temperature, precipitation and soil moisture were demonstrated in PCA analysis, which indicated different patterns of CO_2_ emissions of treatments 20 and 40 cm in the three areas. Moreover, the correlations between CO_2_ fluxes from 20 and 40 cm treatments and soil moisture were directly related to March and April. In this case, one of the largest values of precipitation was recorded in March, while in April, the soil may have had enough moisture to guarantee decomposition. Several studies suggest the highest CO_2_ fluxes occur at the end or beginning of the rainy period, when the porous soil space is also partially filled with air [19, 21, 9, 23, 25, 17]. According to these studies, great water availability can saturate soil pores intervening with the diffusion of gases such as O_2_, used by the bacterial community for decomposition and CO_2_ release. In dry conditions, the osmotic stress of the microbial community of the soil can contribute to the reduction of extracellular enzymatic activity, necessary for decomposing organic substance, leading to a reduction of emissions [62, 9].

The different relationships between soil temperature and moisture variables showed different behaviors in the three areas. Different relationships between temperature and CO_2_ emissions have been presented in literature and the consensus is that it is not possible to define a general pattern for CO_2_ emission controls only by analyzing temperature and soil moisture [16, 17, 19–25]. In this case, temperature and soil moisture are specific to each place and it is too complex to determine which factors contribute to the processing of SOM [25]. According to [9], factors such as climatic conditions (rainfall and droughts), composition of SOM and their behavior by soil characteristics also affect the sensitivity of temperature for decomposition. In general, the temperature sensitivity to CO_2_ emissions is widely reported through Q10 [12, 65–68]. Q10 is defined as the factor by which the rate reaction increases with a 10°C rise in temperature. However, most models are adjusted to temperate regions, and in tropical areas Q10 must be determined for each particular area, which is why Q10 was not used in this study [12].

It is thought that soil texture and management practices in both cacao agroforestry systems (MC and UC) contributed to the different CO_2_ emissions patterns found. The lowest CO_2_ flows in MC were related to the lowest soil moisture influenced by the amount of sandy soil and canopy considered less dense, compared to UC. The soil of MC is more exposed and therefore susceptible to high light intensities. However, in UC, the higher amount of clay and the absence of management to shading control contributes to higher soil moisture. Additionally, in UC, the largest iron concentration and acidity potential H + Al and Fe were 2 and 3 times higher than the values found in MC and PF. In MC, although lower CO_2_ emissions were found, the concentrations of DOC were approximately 3 times higher than those found in PF and UC (*p* < 0.05), and DIC concentrations did not differ from PF.

The three areas have an acid soil with a cation exchange capacity (CEC) exhibiting similar values along the profile. In this case, pH favors biological activity in the entire profiles of the soil in all three areas, [21]. Considering the values of pH and CEC in the three areas it is believed that the CECs in the soil are more influenced by pH than soil texture (e.g. clay) [69]. Chemical attributes such as potential acidity—H+Al, and the addition and saturation of bases—V and SB, confirm that microbial activity is more intense in MC and UC than in PF. PF had the highest organic matter concentration throughout the soil profile, supporting the idea that OM processing is slower than in MC and UC. This corroborates the results found by [62], who compared microbial activity among soils in the Atlantic forest in different treatments. According to these authors, preserved soils are more structured and, therefore, more conducive to protecting organic matter against changes in soil use, resulting in faster adaptations of microbial communities in the soil profile.

According to estimates, managed cacao agroforestry systems can deposit 10 Mg ha^-1^ year^-1^ of plant litter, which creates an important role for the microbial communities to maintain the nutrient cycling [70]. The litter compartment (LD) influenced total CO_2_ fluxes from two agroforestry systems (MC and UC), suggesting different decomposition patterns throughout the soil profile, comparing to PF. Even the depths of 0–20 cm and 20–40 cm exhibited a similar value of soil organic matter (SOM) between UC and PF, the variations of CO_2_ observed between the LD in the different collection areas suggest SOM decomposition in the soil profile releases different components. In MC and UC, the LD values were twice times higher than found in and PF, suggesting the SOM has more complex structural components in MC and UC, thus causing a decomposition that releases greater amount of CO_2_, compared to PF.

The predominance of cacao trees (*Theobroma cacao*) in both systems (MC and UC) may provide a thick litter layer and a greater concentration of roots in the first centimeters of soil compared to PF [71]. The change in composition of SOM in MC and UC can be explained by higher concentrations of nutrients such as Ca^2+^ and Mg^2+^, as well as the dynamics of DOC and DIC in the soil profile. The leaf of *Theobroma cacao* is rich in components that offer physiological and biochemical resistance mechanisms to the attack of some species of fungi and bacteria [72]. Thus, areas with high soil C supply, such as unmanaged cacao agroforestry system have plant litter with modified components [73,74]. Consequently, plant litter from the cacao agroforestry system enriched with nutrients such as P and N [75,76] demands different phases of decomposition that require time intervals, microbial communities and different environmental conditions along the soil layer [22,62,77,78].

In order to make the measurements of CO_2_ fluxes in the F40 treatments, 20 cm of topsoil was removed; therefore, the F40 fluxes are considered artificial. The topsoil removed in the F40 treatment eliminated the "cap" that was preventing upward diffusion of CO_2_, thus allowing CO_2_ to escape into the atmosphere as a CO_2_ efflux surface. When the soil is fully intact, this CO_2_ deeper soil layer most likely remains dissolved and exits the ecosystem via groundwater and hydrological pathways rather than surface emissions. This is supported by a study carried out by [54] at the same study sites, PF and UC.

In all the sites, the higher CO_2_ concentration in the deeper soil layer is due the low rate of CO_2_ production at depth matches the low rates of diffusion of CO_2_ to the surface. As observed in CO_2_, DIC concentrations in the soil profile also tend to increase according to soil depth. The three study areas (PF, MC and UC) have different soil textures, vegetation cover and management that may promote varying CO_2_ diffusion patterns and organic matter transport.

The greater SOM processing in the first soil layers can also be justified by the decrease of DOC concentrations in soil solution and increase of DIC at depths from 15 to 90 cm in the three areas. This pattern is expected, since the first layers depend on the availability of DOC produced by the microbial community, which decrease with depth increases due to sorption of this carbon form [79].

The greater availability of DOC in MC can be attributed to the large amount of organic substrate that was not consumed. Although the soil in this area is classified as Nitosol and Utisol, indicating a layer of higher clay content in deeper soil, the top layer contained 87% sand. In addition to the higher availability of macropores, high sand content promotes better drainage and impairs water retention in the soil, and there is a lower potential for DOC adsorption [80,81]. In this case, difficulties in water retention may not have allowed the soil to reach favorable conditions for activation of microbial activity. Another possible explanation for the higher concentrations of DOC is the rapid processing of OM, which should be incorporated into the microbial network, and the formation of soil aggregates that protect the OM from being decomposed easily.

The potential of the cacao agroforestry system has gained attention due to its high capacity to carbon sequestration in soil maintaining forestry cover [37, 38, 40–44]. However, studies have shown that high concentrations of CO_2_ may alter the soil mineralogy, pH of the groundwater and surface vegetation. These modifications trigger physical and chemical processes, able to modify the properties of the soil [82,83]. Changes in soil use can modify microbial communities and, consequently, ecosystem processes [84–87], and little is known of the effects of great supplies of C in the soil [62]. Another effect of soil with high CO_2_ is the increase of vegetal biomass production, accumulation of metals and different microbial populations associated to the roots, which modify plant-microorganism interactions [88–90]. Thus, to consider that the cacao agroforestry system works as a sink of CO_2_ by storing C in the soil, adequate management that would allow the ecosystemic balance during the processing of SOM is needed. Although this work provides scientific basis to support the idea that cacao agroforestry system in a short time can have a potential of sink to soil-atmosphere CO_2_ emissions, it is still necessary to understand its effects in the long term.

## Conclusions

No differences were found in the total soil CO_2_ fluxes (control fluxes treatments) between the three study areas: preserved forest (PF), managed cacao agroforestry system (MC) and unmanaged cacao agroforestry system (UC). In both cacao agroforestry systems (MC and UC), the total CO_2_ fluxes were influenced by litter decomposition (LD). The soil CO_2_ emissions of the cacao agroforestry systems were highly variable compared to the preserved forest and highly dependent on the soil characteristics attributed to the type of vegetation cover and management. Although a definite pattern between temperature and soil moisture was not observed, these parameters showed a strong relationship with controlling the release of CO_2_ between treatments. The DOC and DIC patterns in the soil solution of the three areas revealed different responses of SOM processing related to soil characteristics and vegetation.

## Supporting information

S1 Table Soil temperature°C. (DOCX)

S2 Table Soil moisture %. (DOCX)

S3 Table CO_2_ fluxes (mg CO_2_—C m^2^ h) of treatments (control, F20 and F40). (DOCX)

S4 Table DOC and DIC (mg L ^-1^) in soil solution. (DOCX)
