# Supplementary material for: Soil organic matter and CO2 fluxes in small tropical watersheds under forest and cacao agroforestry
Source: PLoS One. 2018 Jul 16;13(7):e0200550. doi: 10.1371/journal.pone.0200550 (PMC6047797; doi:10.1371/journal.pone.0200550)
Supplement: S1 Table — (DOCX) [file pone.0200550.s001.docx]

| **Depth** | **Soil temperature °C** | | | | | |
| --- | --- | --- | --- | --- | --- | --- |
|  | **Dec** | **Jan** | **Feb** | **Mar** | **Apr** | **May** |
| **PF** | | | | | | |
| **10 cm** | 27.2 | 26.6 | 27.6 | 29.20 | 26.90 | 30.10 |
|  | 27.4 | 29.1 | 30.9 | 35.30 | 26.70 | 28.30 |
|  | 27.4 | 28.5 | 40.1 | 28.30 | 27.10 | 29.10 |
|  | 30.6 | 27.8 | 28.2 | 26.40 | 27.50 | 26.80 |
|  | 30.5 | 27.8 | 31.4 | 27.70 | 28.10 | 29.70 |
|  | 29.8 | 26.8 | 28.3 | 28.40 |  | 28.4 |
|  |  | 26.60 |  | 29.80 |  | 30.3 |
|  |  | 28.50 |  | 27.10 |  | 28.4 |
|  |  | 28.80 |  | 26.80 |  | 29.6 |
|  |  | 28.00 |  | 27.80 |  | 29.4 |
|  |  | 26.3 |  | 28.10 |  |  |
|  |  | 28 |  | 27.40 |  |  |
|  |  |  |  | 30.20 |  |  |
|  |  |  |  | 27.60 |  |  |
| **20 cm** | 27.2 | 26.1 | 31.4 | 27.9 | 27.9 | 28.3 |
|  | 27.20 | 27.4 | 29.80 | 27.1 | 27.1 | 26.3 |
|  | 30.60 | 27.8 | 28.30 | 27.8 | 27.8 | 28.1 |
|  | 28.70 | 26.5 |  | 27.1 | 27.1 | 27.2 |
|  |  | 28.9 |  | 27.3 |  | 27.7 |
|  |  | 26.0 |  | 28.7 |  | 28.1 |
|  |  | 25.8 |  | 28.3 |  | 28.5 |
|  |  | 26.6 |  | 27.6 |  | 29.2 |
|  |  |  |  | 26.9 |  |  |
|  |  |  |  | 27.8 |  |  |
| **MC** | | | | | | |
| **10 cm** | 27.60 | 25.8 | 25.4 | 26.80 | 25.90 | 25.80 |
|  | 24.50 | 26.6 | 28.7 | 31.30 | 26.20 | 25.40 |
|  | 29.10 | 27.8 | 26.6 | 26.50 | 27.20 | 23.20 |
|  | 28.00 | 26.7 | 26.7 | 27.90 | 27.10 | 25.40 |
|  | 26.2 | 26.6 |  | 32.40 | 26.60 | 25.60 |
|  | 28.2 | 29.3 |  | 25.90 | 26.2 | 24.8 |
|  | 22.5 | 24.7 |  | 26.70 |  |  |
|  | 29.4 | 24.6 |  | 27.60 |  |  |
|  | 24.9 | 25.7 |  | 26.5 |  |  |
|  | 22.30 | 25.00 |  | 28.00 |  |  |
|  |  | 25.40 |  | 26.10 |  |  |
|  |  | 25.60 |  | 26.90 |  |  |
|  |  |  |  | 26.90 |  |  |
|  |  |  |  | 25.80 |  |  |
|  |  |  |  | 25.80 |  |  |
|  |  |  |  | 25.10 |  |  |
| **20 cm** | 25.5 | 26.40 | 27.5 | 26.7 | 26.50 | 24.6 |
|  | 25.8 | 26.8 | 28.6 | 30.1 | 29.20 | 25.1 |
|  | 28.6 | 26.7 | 27.1 | 26.1 | 27.40 | 25.2 |
|  | 27.8 | 29.4 | 27.9 | 28.7 | 26.30 | 25.4 |
|  | 28.60 | 24.30 |  | 27.30 |  | 25.90 |
|  | 25.60 | 25.30 |  | 27.80 |  | 25.20 |
|  | 25.50 | 24.90 |  | 26.40 |  | 24.80 |
|  | 26.20 |  |  | 26.80 |  |  |
|  |  |  |  | 28.20 |  |  |
|  |  |  |  | 26.60 |  |  |
|  |  |  |  | 26.9 |  |  |
| **UC** | | | | | | |
| **10 cm** | 27.5 | 25.7 | 26.1 | 26.9 | 26.70 | 26.20 |
|  | 25.4 | 25.5 | 25.9 | 27.3 | 27.70 | 26.80 |
|  | 25.9 | 25.7 | 26.5 | 28.7 | 27.10 | 27.10 |
|  | 26.90 | 26.20 | 27.30 | 26.70 | 27.20 | 27.70 |
|  | 26.50 | 25.80 | 27.20 | 27.40 | 27.00 | 27.10 |
|  | 25.70 | 25.30 | 26.50 | 27.20 | 27.00 | 26.90 |
|  |  | 25.10 |  | 27.40 | 26.10 | 27.20 |
|  |  | 25.00 |  | 27.20 | 26.40 | 27.10 |
|  |  | 25.00 |  |  | 26.20 | 27.10 |
|  |  | 25.10 |  |  | 26.10 | 26.80 |
|  |  | 25.20 |  |  | 26.20 | 26.70 |
|  |  |  |  |  | 28.00 | 27.30 |
|  |  |  |  |  | 28.2 |  |
|  |  |  |  |  | 28.0 |  |
| **20 cm** | 25.03 | 25.4 | 25.9 | 28.1 | 26.9 | 26.2 |
|  | 25.3 | 25.4 | 26.8 | 28.8 | 26.4 | 27 |
|  | 26.5 | 26.1 | 27.3 | 28.4 | 27 | 27.1 |
|  | 26.1 | 25.8 | 26.5 | 27.2 | 26.9 | 27.1 |
|  | 25.3 | 25.1 | 26.5 | 28.8 | 26.4 | 26.2 |
|  |  | 25.1 |  | 27.2 | 27 | 26.2 |
|  |  | 25.1 |  |  | 26.2 | 27.3 |
|  |  | 25.1 |  |  | 26.1 |  |
|  |  |  |  |  | 26.2 |  |
|  |  |  |  |  | 26.1 |  |
|  |  |  |  |  | 26.3 |  |
|  |  |  |  |  | 27.9 |  |
