# Supplementary material for: Soil organic matter and CO2 fluxes in small tropical watersheds under forest and cacao agroforestry
Source: PLoS One. 2018 Jul 16;13(7):e0200550. doi: 10.1371/journal.pone.0200550 (PMC6047797; doi:10.1371/journal.pone.0200550)
Supplement: S2 Table — (DOCX) [file pone.0200550.s002.docx]

| **Depth** | **Soil moisture %** | | | | | |
| --- | --- | --- | --- | --- | --- | --- |
|  | **Dec** | **Jan** | **Feb** | **Mar** | **Apr** | **May** |
| **PF** | | | | | | |
| **10 cm** | 30.5 | 32.1 | 31.0 | 31.20 | 37.51 | 35.88 |
|  | 29.8 | 32.3 | 31.5 | 34.79 | 34.30 | 36.92 |
|  | 30.9 | 35.3 | 31.3 | 33.93 | 34.30 | 35.35 |
|  | 29.5 | 34.9 |  | 32.22 | 37.02 | 35.38 |
|  | 30.4 | 35.5 |  | 33.17 | 36.74 |  |
|  | 30.8 | 33.8 |  | 32.78 | 38.69 |  |
|  |  |  |  | 34.74 |  |  |
| **20 cm** | 30.83 | 33.99 | 31.96 | 34.52 | 33.51 | 36.08 |
|  | 31.20 | 32.60 | 31.49 | 32.64 | 34.16 | 35.66 |
|  | 29.80 | 31.80 | 31.12 | 32.48 | 39.94 | 36.17 |
|  |  |  |  | 32.54 | 38.25 | 36.41 |
|  |  |  |  | 32.26 | 35.43 |  |
|  |  |  |  | 33.65 |  |  |
|  |  |  |  | 32.74 |  |  |
| **MC** | | | | | | |
| **10 cm** | 3.39 | 16.48 | 11.00 | 8.21 | 12.09 | 14.39 |
|  | 5.06 | 12.47 | 0.43 | 8.94 | 9.19 | 13.39 |
|  | 6.15 | 11.27 | 3.94 | 10.69 | 8.92 | 14.04 |
|  | 7.61 | 13.86 |  | 14.96 |  | 15.55 |
|  | 4.83 | 13.64 |  | 9.58 |  | 18.29 |
|  | 4.59 | 11.34 |  | 11.21 |  | 20.16 |
|  |  |  |  | 6.03 |  |  |
|  |  |  |  | 6.39 |  |  |
|  |  |  |  | 6.32 |  |  |
| **20 cm** | 3.46 | 9.12 | 7.38 | 7.31 | 8.61 | 11.49 |
|  | 4.21 | 10.5 | 5.45 | 8.54 | 10.00 | 13.93 |
|  | 4.10 | 9.34 | 8.73 | 10.49 | 9.76 | 12.59 |
|  | 3.46 |  |  | 11.70 |  | 13.87 |
|  | 3.32 |  |  | 9.14 |  | 16.88 |
|  |  |  |  | 10.89 |  | 16.33 |
|  |  |  |  | 7.30 |  |  |
|  |  |  |  | 7.74 |  |  |
|  |  |  |  | 5.64 |  |  |
| **UC** | | | | | | |
| **10 cm** | 12.89 | 26.16 | 21.33 | 15.74 | 16.47 | 34.69 |
|  | 17.32 | 24.60 | 15.95 | 23.74 | 23.03 | 27.40 |
|  | 11.61 | 21.39 | 22.20 | 13.15 | 25.17 | 26.02 |
|  | 17.45 | 26.45 |  | 21.34 | 23.84 | 34.91 |
|  | 11.32 | 25.52 |  | 24.75 | 23.37 | 34.67 |
|  |  | 21.37 |  | 25.74 | 17.77 | 41.19 |
|  |  | 25.89 |  | 24.42 | 27.39 | 34.17 |
|  |  | 21.68 |  |  | 34.61 |  |
|  |  |  |  |  | 33.16 |  |
|  |  |  |  |  | 43.53 |  |
|  |  |  |  |  | 34.20 |  |
| **20 cm** | 16.39 | 20.50 | 19.44 | 22.80 | 30.87 | 24.01 |
|  | 18.17 | 23.50 | 15.72 | 22.46 | 18.44 | 36.22 |
|  | 12.50 | 22.45 |  | 22.82 | 21.67 | 28.39 |
|  | 12.67 |  |  | 20.99 | 25.51 | 34.91 |
|  |  |  |  | 20.62 | 24.98 | 34.67 |
|  |  |  |  | 20.60 | 24.35 | 41.19 |
|  |  |  |  |  | 19.04 | 34.17 |
|  |  |  |  |  | 31.47 | 40.79 |
|  |  |  |  |  | 39.13 | 37.54 |
|  |  |  |  |  | 35.59 | 38.35 |
|  |  |  |  |  | 32.44 | 37.57 |
