# Supplementary material for: Soil organic matter and CO2 fluxes in small tropical watersheds under forest and cacao agroforestry
Source: PLoS One. 2018 Jul 16;13(7):e0200550. doi: 10.1371/journal.pone.0200550 (PMC6047797; doi:10.1371/journal.pone.0200550)
Supplement: S3 Table — (DOCX) [file pone.0200550.s003.docx]

| **Treatment** | **mg CO_2_ - C m^2^ h** | | | | | |
| --- | --- | --- | --- | --- | --- | --- |
|  | **Dec** | **Jan** | **Feb** | **Mar** | **Apr** | **May** |
| **PF** | | | | | | |
| **CF** | 1.70 | 2.34 | 50.53 | 66.64 | 98.53 | 48.6 |
|  | 1.70 | 1.89 | 6.31 | 72.44 | 112.30 | 30.7 |
|  |  | 5.74 |  | 100.22 |  |  |
|  |  | 5.85 |  | 63.79 |  |  |
| **F20** | 3.74 | 2.09 | 51.53 | 29.60 | 229.36 | 16.86 |
|  | 3.07 | 2.47 | 7.43 | 14.72 | 231.54 | 104.39 |
|  | 2.68 | 6.19 | 12.88 | 112.61 | 171.63 | 66.38 |
|  | 3.04 | 4.61 | 26.90 | 192.39 |  | 187.74 |
|  |  | 13.98 |  | 247.22 |  | 16.61 |
|  |  | 3.59 |  | 161.26 |  | 61.68 |
|  |  | 6.48 |  | 214.31 |  | 72.97 |
|  |  | 4.09 |  | 194.61 |  | 13.48 |
|  |  |  |  | 367.37 |  |  |
| **F40** | 6.21 | 4.91 | 17.23 | 27.98 | 231.91 | 57.98 |
|  | 12.41 | 17.18 | 45.32 | 69.80 | 569.98 | 63.77 |
|  | 4.12 | 6.11 | 32.33 | 19.39 | 577.54 | 603.91 |
|  | 7.45 | 3.53 | 7.92 | 44.36 | 353.39 | 361.52 |
|  |  | 4.36 |  | 158.08 |  | 267.97 |
|  |  | 9.23 |  | 440.47 |  | 93.06 |
|  |  | 4.59 |  | 378.69 |  | 138.87 |
|  |  | 6.14 |  | 184.13 |  | 195.06 |
|  |  |  |  | 464.11 |  |  |
|  |  |  |  | 447.14 |  |  |
|  |  |  |  | 441.42 |  |  |
| **MC** | | | | | | |
| **CF** | 2.42 | 1.74 | 3.16 | 16.80 | 64.66 | 18.96 |
|  | 1.33 | 1.20 | 3.16 | 112.46 | 85.88 | 78.80 |
|  | 1.88 | 2.78 |  | 185.19 |  | 4.10 |
|  | 2.06 | 2.34 |  | 84.87 |  | 101.11 |
|  |  |  |  | 126.83 |  |  |
| **F20** | 0.55 | 2.45 | 4.93 | 20.86 | 21.33 | 14.52 |
|  | 0.98 | 2.11 | 5.02 | 12.66 | 54.31 | 28.56 |
|  | 2.02 | 1.30 | 3.75 | 18.80 | 75.45 | 82.67 |
|  | 2.41 | 1.28 |  | 9.12 | 44.52 | 20.51 |
|  | 5.04 | 1.76 |  | 42.87 |  | 7.08 |
|  | 0.98 | 2.37 |  | 91.22 |  |  |
|  | 1.20 | 2.34 |  | 107.28 |  |  |
|  |  | 1.36 |  | 29.01 |  |  |
|  |  |  |  | 109.66 |  |  |
|  |  |  |  | 50.58 |  |  |
|  |  |  |  | 40.08 |  |  |
| **F40** | 8.47 | 8.58 | 102.40 | 127.63 | 141.06 | 51.53 |
|  | 2.96 | 4.91 | 5.12 | 19.06 | 308.15 | 73.70 |
|  | 13.43 | 10.02 | 13.96 | 27.23 | 275.52 | 182.74 |
|  | 8.63 | 5.38 | 5.16 | 14.82 | 101.27 | 65.32 |
|  | 5.88 | 4.53 |  | 140.95 |  | 65.97 |
|  | 8.07 | 4.57 |  | 386.65 |  | 50.53 |
|  | 0.13 | 3.59 |  | 184.47 |  | 57.47 |
|  | 1.93 |  |  | 32.90 |  |  |
|  | 11.18 |  |  | 195.21 |  |  |
|  |  |  |  | 251.72 |  |  |
|  |  |  |  | 318.54 |  |  |
|  |  |  |  | 188.81 |  |  |
| **UC** | | | | | | |
| **CF** | 6.98 | 6.99 | 11.40 | 77.05 | 235.06 | 69.68 |
|  | 10.31 | 4.62 | 5.86 | 1078.48 | 315.42 | 179.49 |
|  |  | 5.02 |  |  | 112.22 | 54.51 |
|  |  |  |  |  | 163.09 | 80.01 |
|  |  |  |  |  |  |  |
| **F20** | 8.17 | 3.42 | 14.72 | 16.83 | 273.12 | 116.95 |
|  | 6.24 | 4.08 | 6.78 | 28.03 | 250.74 | 67.57 |
|  | 4.31 | 5.27 | 7.18 | 206.81 | 56.91 | 103.18 |
|  | 9.72 | 7.83 | 19.05 |  | 494.50 | 27.21 |
|  |  | 10.04 |  |  | 174.99 | 54.65 |
|  |  | 11.61 |  |  | 292.79 | 79.36 |
|  |  | 16.55 |  |  | 48.77 | 61.66 |
|  |  | 3.51 |  |  | 32.13 | 48.54 |
|  |  |  |  |  | 5.80 |  |
|  |  |  |  |  |  |  |
|  |  |  |  |  |  |  |
| **F40** | 23.45 | 68.21 | 89.39 | 100.43 | 1671.57 | 1169.72 |
|  | 29.73 | 30.86 | 221.23 | 3858.49 | 1518.41 | 776.33 |
|  | 24.00 | 50.70 | 382.77 | 426.07 | 788.15 | 971.47 |
|  | 20.91 | 11.01 | 61.02 | 1535.91 | 1690.76 | 634.54 |
|  |  | 5.21 |  | 702.61 | 896.88 | 171.84 |
|  |  | 9.09 |  |  | 1672.58 | 115.45 |
|  |  | 9.75 |  |  | 155.00 | 1668.91 |
|  |  | 5.62 |  |  | 251.43 | 2000.81 |
|  |  |  |  |  | 101.32 |  |
|  |  |  |  |  | 419.86 |  |
