# Supplementary material for: Soil organic matter and CO2 fluxes in small tropical watersheds under forest and cacao agroforestry
Source: PLoS One. 2018 Jul 16;13(7):e0200550. doi: 10.1371/journal.pone.0200550 (PMC6047797; doi:10.1371/journal.pone.0200550)
Supplement: S4 Table — (DOCX) [file pone.0200550.s004.docx]

| **Site** | **DOC mg L ^-1^** | | | **DIC mg L ^-1^** | | |
| --- | --- | --- | --- | --- | --- | --- |
|  | **10 cm** | **45 cm** | **90 cm** | **10 cm** | **45 cm** | **90 cm** |
| **PF** | 7.74 | 9.05 | 6.67 | 0.85 | 0.77 | 2.57 |
|  | 3.54 | 10.26 | 6.83 | 1.06 | 0.82 | 1.28 |
|  | 5.87 | 9.61 | 2.77 | 0.51 | 0.74 | 1.52 |
|  | 2.36 | 5.61 | 1.10 | 0.52 | 1.20 | 7.07 |
|  | 20.71 | 2.02 | 8.88 | 0.59 | 0.60 | 6.70 |
|  | 15.87 | 2.23 | 10.25 | 0.57 | 1.28 | 0.50 |
|  | 27.04 | 1.71 | 2.94 | 0.52 | 1.97 | 0.54 |
|  | 17.51 | 1.84 | 2.54 | 0.52 | 1.64 | 0.53 |
|  |  | 6.33 | 2.78 |  | 0.49 | 0.53 |
|  |  | 4.16 | 36.21 |  | 0.62 | 0.54 |
|  |  | 2.81 | 7.58 |  | 0.50 | 0.54 |
|  |  | 4.13 | 5.64 |  | 0.56 | 0.50 |
|  |  | 12.58 | 20.89 |  | 0.50 | 0.50 |
|  |  | 10.10 |  |  | 0.53 |  |
|  |  | 13.25 |  |  | 0.53 |  |
|  |  | 12.71 |  |  | 0.56 |  |
| **MC** | 37.76 | 42.35 | 26.63 | 0.91 | 3.61 | 1.97 |
|  | 47.42 | 20.55 | 16.51 | 1.52 | 3.64 | 1.01 |
|  | 72.06 | 67.85 | 17.60 | 1.94 | 2.86 | 1.99 |
|  | 64.87 | 39.86 | 4.96 | 0.79 | 1.30 | 6.07 |
|  | 54.17 | 37.17 | 20.96 | 0.61 | 1.32 | 1.98 |
|  | 26.10 | 16.87 | 20.10 | 1.54 | 1.49 | 2.02 |
|  |  | 33.71 | 9.70 |  | 5.28 | 5.37 |
|  |  | 9.98 | 16.65 |  | 3.63 | 4.11 |
|  |  | 21.70 | 12.58 |  | 1.68 | 3.34 |
| **UC** | 14.20 | 11.08 | 6.21 | 0.49 | 0.89 | 3.38 |
|  | 7.24 | 0.77 | 4.04 | 0.52 | 6.36 | 6.91 |
|  | 16.95 | 3.10 | 5.61 | 0.53 | 3.60 | 11.35 |
|  | 8.89 | 4.95 | 11.91 | 0.49 | 3.55 | 2.88 |
|  | 6.56 | 4.01 | 6.50 | 0.52 | 3.55 | 6.17 |
